# Effect of Cold-Rolling Reduction on Recrystallization Microstructure, Texture and Corrosion Properties of the X2CrNi12 Ferritic Stainless Steel

**DOI:** 10.3390/ma15196914

**Published:** 2022-10-05

**Authors:** Rui Li, Binguo Fu, Yufeng Wang, Jingkun Li, Tianshun Dong, Guolu Li, Guixian Zhang, Jinhai Liu

**Affiliations:** 1 Department of Materials Processing and Control Engineering, School of Materials Science and Engineering, Hebei University of Technology, Tianjin 300401, China; 2 Aerospace Product Center, Tianjin Institute of Aerospace Mechanical and Electrical Equipment, Tianjin 300301, China; 3 Key Laboratory of Research and Application of Mould Materials for Glass and Rubber in Hebei Province, Hebei Andi Mould Co., Ltd., Huanghua 061100, China

**Keywords:** ferritic stainless steel, microstructure, texture, grain boundary engineering, corrosion resistance

## Abstract

X2CrNi12 ferritic stainless steel has a wide range of application prospects in the railway transportation, construction, and automobile fields due to its excellent properties. The properties of X2CrNi12 ferritic stainless steel can be further improved by cold-rolling and subsequent annealing treatment. The purpose of this work is to investigate the effect of cold-rolling reduction on the microstructure, texture and corrosion properties of the recrystallized X2CrNi12 ferritic stainless steel by using SEM, TEM, EBSD and electrochemical testing technology. The results show that the crystal orientation characteristics of the cold-rolled sheet could be inherited into the annealed sheet. The higher cold-rolling reduction could promote the deformed grains rotating into the {111}<uvw> orientation, increasing storage energy and driving force for recrystallization, which could reduce the recrystallized grain size. The orientation densities of α-fiber and γ-fiber were low at 50% cold-rolling reduction. After recrystallization annealing, a large number of grains with random orientation could be produced, and the texture strength was weakened. When the cold-rolling reduction rose to 90%, the γ-fiber texture at {111}<110> was strengthened and the α-fibers, particularly the {112}<110> component, were weakened after recrystallisation annealing, which could improve the formability of the steels. The proportions of special boundaries, i.e., low-angle grain boundaries and low-Σ CSL boundaries, among the grain boundary distribution of the recrystallized X2CrNi12 stainless steel were higher when the reduction was 90%, especially when the annealing temperature was 770 °C. Additionally, the proportion of LAGBs and low-Σ CSL boundaries were 53% and 7.43%, respectively, which improves the corrosion resistance of the matrix, showing the best corrosion resistance.

## 1. Introduction

As a kind of prospective low chromium ferritic stainless steel, X2CrNi12 is inexpensive compared to austenitic stainless steels and medium-high chromium ferritic stainless steels, while it shows excellent corrosion resistance compared with carbon steels and weathering steels [1,2]. Besides, it has brought increased applications in railway transportation, building structures and automobile exhaust systems with its excellent weldability and mechanical properties [3,4,5]. However, the lower Cr in the matrix leads to the poor corrosion property of X2CrNi12 compared to austenitic stainless steel and medium- and high-chromium ferritic stainless steels, and its application is restricted in environments requiring high corrosion resistance. In addition, the formability of the ferritic stainless steels is limited compared to the austenitic and low-carbon steels, which leads to easy thinning during deep drawing, which is not conductive to the degree of deep drawing deformation and product surface quality. Therefore, it is of great significance to carry out extensive work to improve the corrosion performance and formability of X2CrNi12 ferritic stainless steel.

In our previous work, the effects of annealing temperature on the microstructure, mechanical property and anticorrosion behavior of X2CrNi12 ferritic stainless steel were systematically investigated [6]. The precipitation behavior of (Fe, Cr)_23_C_6_ carbides and martensite and their effects on mechanical and corrosion properties were revealed. However, the formability was not involved. The formability of ferritic stainless steel closely depends on the texture evolution during the annealing process, and the strong γ-fiber (<111>//ND) texture could improve the formability of the stainless steels [7,8,9]. Abreu et al. [10] studied AISI-444 ferritic stainless steel and found that high temperature annealing was beneficial to improve the strength of γ-fiber texture at {111}<112> and reduce the surface ridging. Huh et al. [11] introduced intermediate annealing after cold-rolling, which led to a weaker rolling texture in the bcc metals, which had less pronounced texture evolution. Thus, the recrystallization annealing process could promote the typical γ-fiber texture, improving the formability of the steel. Zhang et al. [12] claimed that the existence of shear bands could enhance the recrystallization nucleation rate during the hot-rolling and annealing process, which intensified the {111} textures in the final sheet. Rodrigues et al. [13] pointed out that reducing the size of the original grains of the specimen can enhance the γ-fiber component among the recrystallization textures. In addition, the distribution of the grain boundary has a great influence on the mechanical and corrosion properties of materials [14,15,16,17]. Yan et al. [14] found that the fractions of the Σ3 and Σ13b grain boundaries of Nb + Ti-stabilized ferritic stainless steel increased by two-step cold-rolling and annealing treatment, which improved the corrosion resistance of stainless steel. Han et al. [15] reported that adding low-Σ CSLs, e.g., Σ1, Σ3, and 2-CSL and 3-CSL triple junctions, through breaking up the connectivity of the random boundary networks, could improve the mechanical properties of materials. Shimada et al. [16] increased the proportion of grain boundaries with Σ ≤ 29 to make the material have high resistance to intergranular corrosion.

It can be seen that rolling and annealing treatments have important effects on microstructure evolution and properties. However, the effect of cold-rolling reduction on the recrystallization structure, texture evolution, grain boundary distribution and corrosion resistance of low Cr ferritic stainless steel is rarely studied, especially for X2CrNi12 ferritic stainless steel. In this paper, the microstructure, texture evolution and grain boundary distribution of different recrystallization-annealed specimens with 50% and 90% cold-rolling reductions were systematically studied, and the corresponding corrosion properties were analyzed. This has important theoretical and practical significance to improve corrosion resistance and expand the range of applications.

## 2. Materials and Methods

### 2.1. Materials and Processing

The material used was commercial hot-rolled X2CrNi12 ferritic stainless steel plates with a thickness of 6.2 mm; the composition is shown in Table 1. It was cold-rolled to a thickness of 3.2 mm and 0.46 mm on a Φ200 cold-rolling mill with hydraulic tension, with a corresponding reduction of 50% and 90%, respectively. Then, the cold-rolled sheets were heated to 720 °C, 740 °C and 770 °C for 30 min, followed by air cooling. The specimens were expressed as 50%-deformed, 50%-720 °C, 50%-740 °C, 50%-770 °C, 90%-deformed, 90%-720 °C, 90%-740 °C and 90%-770 °C, respectively.

### 2.2. Microstructure Characterization

The microstructures of specimens were observed by a JSM-6510 scanning electron microscope (SEM, JEOL, Tokyo, Japan) with an accelerating voltage of 20 kV and a Tecnai G2 F30 transmission electron microscope (TEM, FEI, Amsterdam, The Netherlands). The SEM specimens were ground, polished and then etched by 3 mL HNO_3_ + 9 mL HCl + 12 mL H_2_O. The TEM specimens were ground to 50 μm, punched to Φ3 mm discs and ion-milled. To further analyze the textures and microstructures, the rolling surface as defined by rolling direction (RD) and transverse direction (TD) were studied by electron backscattered diffraction (EBSD). Specimens were electro-polished by an applied potential of 30 V with 5% perchloric acid + 95% alcohol at −25 °C for 60 s. EBSD measurements were carried out by using the JSM-7200F field emission scanning electron microscope (FESEM, JEOL, Tokyo, Japan) with EBSD detector. The step of EBSD was 0.5 µm, and the EBSD data were analyzed by using the Channel 5 software (Oxford Instruments, Oxford, UK). The low-angle grain boundaries (LAGBs) were defined as 2° ≤ θ ≤ 15°, while the high-angle grain boundaries (HAGBs) were defined as θ > 15°.

### 2.3. Electrochemical Test

The surfaces of the test specimens were sanded with SiC sandpaper to 2000#, and then polished and ultrasonically cleaned in alcohol. Before the electrochemical test, the specimens were encapsulated in epoxy resin; the test surface was composed of RD and TD, leaving a 1 cm^2^ working surface. The electrochemical test was performed by using a CHI660D three-electrode electrochemical workstation. The counter electrode (CE) was the Pt electrode, the reference electrode (RE) was the saturated calomel electrode (SCE), and the working electrode (WE) was the specimen. Since the stainless steel is prone to localized corrosion in solutions containing Cl^−^, such as marine environments, a 3.5 wt.% NaCl electrolyte was used to obtain the marine environment and to have a deeper understanding of the actual corrosion of the alloy in the real working environment.

The tested specimen (working electrode) was first immersed in the 3.5 wt.% NaCl solution for 1800 s to reach a stable open circuit potential (OCP). The potentiodynamic polarization test was then started. The potential changed from −1.5 V to 0.5 V, and the scan rate of the polarization test was 1 mV/s. The scan frequency of electrochemical impedance spectroscopy (EIS) was 10^−2^ Hz–10^5^ Hz, and the disturbance amplitude was 10 mV. The ZsimpWin V3.61 software (AMETEK Scientific Instruments, Berwyn, IL, USA) was used to analyze the impedance data. In order to ensure the accuracy of the experiment, three specimens were measured at each condition.

## 3. Results and Discussion

### 3.1. Microstructure

**Figure 1:**
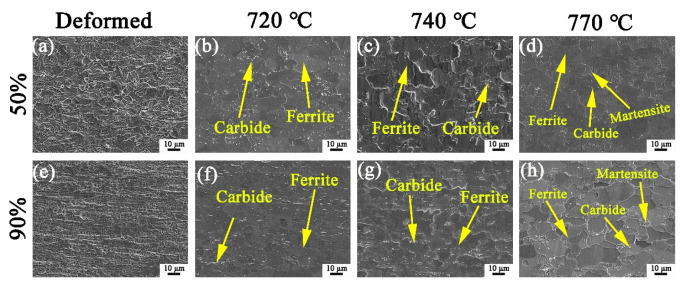
Microstructures of X2CrNi12 steel at 50% deformation (**a**–**d**) and 90% deformation (**e**–**h**) with different annealing temperatures, (**a**,**e**) deformed; (**b**,**f**) 720 °C; (**c**,**g**) 740 °C; (**d**,**h**) 770 °C.

**Figure 2:**
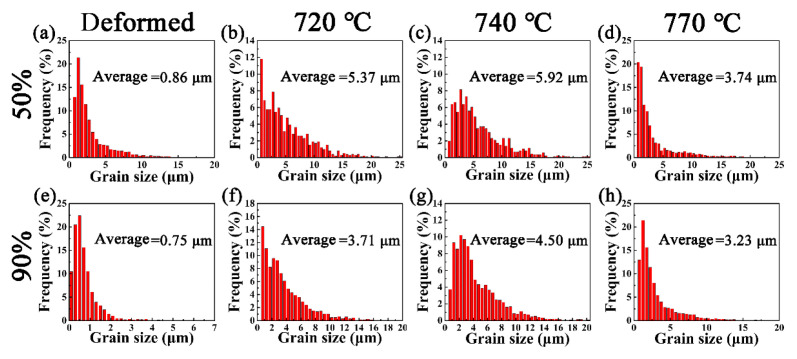
Grain size distribution of X2CrNi12 steel at 50% deformation (**a**–**d**) and 90% deformation (**e**–**h**) with different annealing temperatures, (**a**,**e**) deformed; (**b**,**f**) 720 °C; (**c**,**g**) 740 °C; (**d**,**h**) 770 °C.

Figure 1 and Figure 2 show the microstructures and related grain size distribution of the cold-rolled stainless steel with 50% and 90% reductions, followed by annealing treatment at 720 °C, 740 °C and 770 °C, respectively. When the cold-rolling reduction is 50%, the microstructure is mainly ferrite and some grains are elongated and broken along the rolling direction, with an average grain size of 0.86 μm (Figure 1a and Figure 2a). When the cold-rolling reduction is 90%, the grains are further broken, and the grains are severely elongated to form a fibrous structure. The average grain size is only 0.75 μm (Figure 1e and Figure 2e). After annealing at 720 °C for 30 min, the ferrite grains were basically recrystallized, and a large number of white precipitates precipitated on the matrix (Figure 1b,f). The average grain size of the specimens at 50%-720 °C and 90%-720 °C increased to 5.37 μm and 3.71 μm, respectively (Figure 2b,f). When the annealing temperature is 740 °C, it can be seen that a large number of white precipitates further precipitate on the substrate. Compared with the specimens annealed at 720 °C for 30 min, the ferrite grains grew (Figure 1c,g), and the average grain size of the specimens at 50%-740 °C and 90%-740 °C increased to 5.92 μm and 4.50 μm, respectively (Figure 2c,g). As shown in Figure 1d,h, after annealing at 770 °C for 30 min, the number of precipitates is significantly reduced, and some grains have transformed into martensite through martensitic transformation. Additionally, the specimens consisted of ferrite, martensite and a small number of precipitated phases. It is worth noting that there are almost no precipitates on the ferrite grain boundaries, and a few precipitates are located on the martensite, which may be caused by both the segregation of Cr and the supersaturation of C in martensite [18,19]. With the increase of temperature, the ferrite grains increase and become equiaxed gradually, but the average grain size of the 50%-770 °C and 90%-770 °C specimens reduced to 3.74 μm and 3.23 μm, respectively, due to the generation of small size of martensite (Figure 2d,h). It can be seen that the recrystallized grains of the specimen with 90% cold-rolling reduction are smaller than those at 50% cold-rolling reduction. The severe grain fragmentation under large deformation provides more recrystallization nucleation sites, and the large storage energy in the specimen under large deformation can promote the nucleation rate [12].

**Figure 3:**
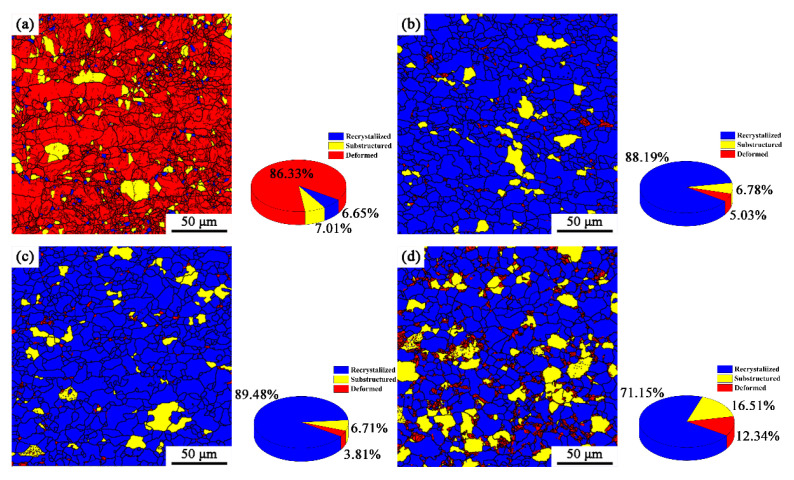
Recrystallization microstructure distribution of X2CrNi12 steel at different annealing temperatures with 50% cold-rolling deformation (**a**) deformed; (**b**) 720 °C; (**c**) 740 °C; (**d**) 770 °C.

**Figure 4:**
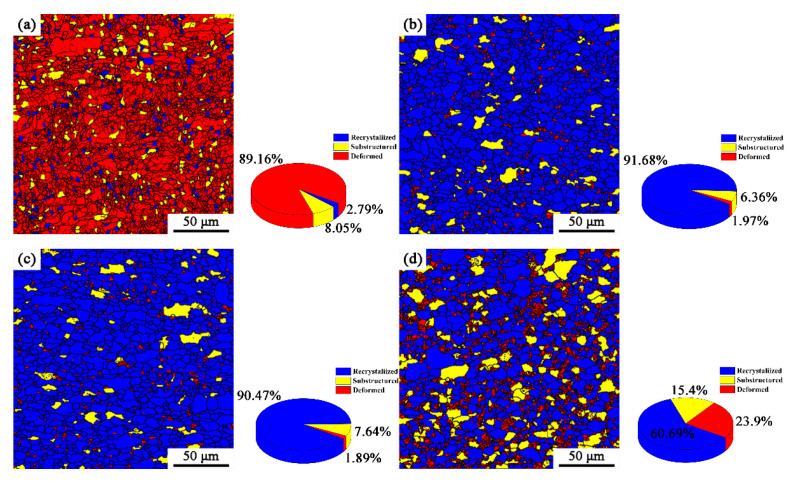
Recrystallization microstructure distribution of X2CrNi12 steel at different annealing temperatures with 90% cold-rolling deformation (**a**) deformed; (**b**) 720 °C; (**c**) 740 °C; (**d**) 770 °C.

The recrystallization microstructure of the specimens with annealing at different temperatures after 50% and 90% cold-rolling are shown in Figure 3 and Figure 4. According to the difference in the misorientation angles between the adjacent grains, the red, yellow and blue regions are deformed grains (LAGBs), ferrite subgrains (Middle) and recrystallized grains (HAGBs), respectively. It can be seen from Figure 3 that when the cold-rolling reduction is 50%, the grain is flattened and deformed, and part of the grain has been broken, with a deformed grain proportion of 86.33% (Figure 3a). When the annealing temperature is 720 °C, the ferrite grains were basically recrystallized (Figure 3b). With the increase of annealing temperature, the recrystallization quantity does not change much compared with that at 720 °C, but the grain size increases (Figure 3c). It is worth noting that when the annealing temperature was 770 °C, the recrystallization region decreased, and the deformed region increased greatly, which is mainly because of the partial martensitic transformation that occurred. The higher lattice distortion energy in the plastic deformation zone provides sites for martensite nucleation and promotes nucleation rate. Therefore, there are more martensite subgrain boundaries in the deformation region. In addition, the increase of annealing temperature will cause the growth of ferrite subgrains, and then increase its volume fraction. From Figure 4, it can be seen that a cold-rolling reduction of 90% increases the number of deformed grains and ferrite subgrains in the steels, and their contents are 89.16% and 8.05%, respectively. When the annealing temperature is 720 °C and 740 °C, the recrystallization is basically completed, and the amount of recrystallization is slightly higher than that at 50%-720 °C and 50%-740 °C (Figure 4b,c). When the annealing temperature was 770 °C, the ferrite grains in the matrix recrystallized completely (Figure 4d), and the deformation regions (martensite subgrains) and the volume fraction of ferrite subgrains increased more obviously compared with that of the 50% reduction, which indicates that when the annealing of X2CrNi12 stainless steel enters the (Austenite + Ferrite) two phases region, the large cold-rolling reduction can promote the formation of martensite [6].

**Figure 5:**
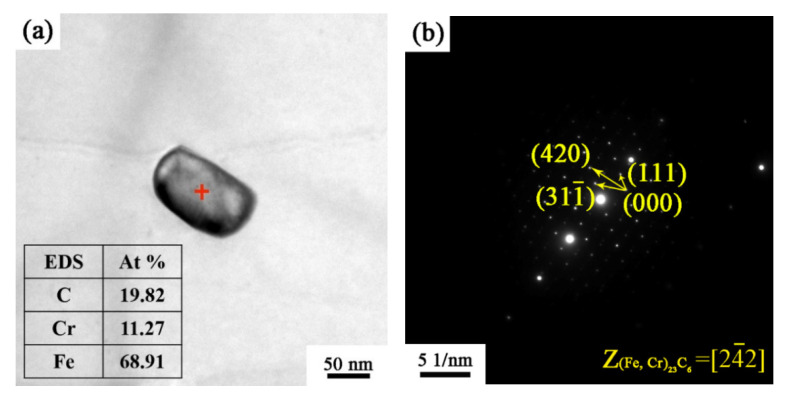
(**a**) Bright-field TEM images showing the (Fe, Cr)_23_C_6_ precipitated from the as-annealed X2CrNi12 ferritic stainless steel and (**b**) the corresponding SAED pattern.

To display the microstructure and composition of the precipitates more clearly, Figure 5a shows the TEM image and the corresponding EDS of the precipitates in the specimen annealed at 740 °C. It can be seen that the precipitate was mainly composed of Fe, Cr, and C elements. Figure 5b shows the selected area electron diffraction (SAED) pattens of the precipitate in Figure 5a. The EDS result and diffraction pattern provide evidence for the precipitation of (Fe, Cr)_23_C_6_, and display a face-centered cubic crystal structure with a lattice constant of 0.106 nm.

### 3.2. Texture Evolution

**Figure 6:**
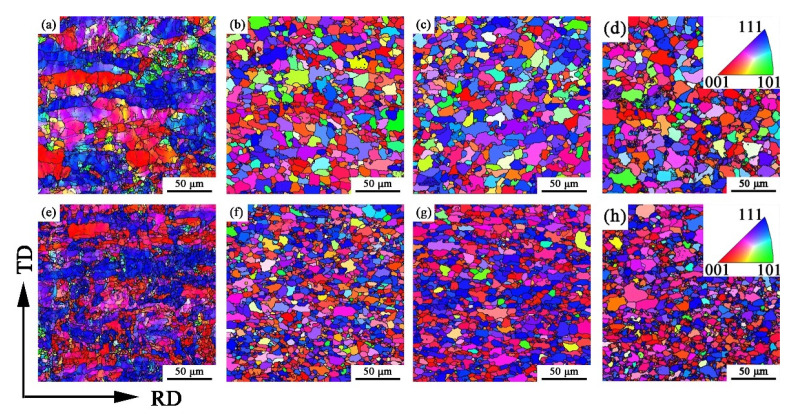
Inverse pole figure (IPF) orientation map projected from the normal of the specimen surface at 50% deformation (**a**–**d**) and 90% deformation (**e**–**h**) with different annealing temperatures, (**a**,**e**) deformed; (**b**,**f**) 720 °C; (**c**,**g**) 740 °C; (**d**,**h**) 770 °C.

The orientation maps of the specimens obtained by EBSD at 50% and 90% cold-rolling reduction followed by annealing treatment with different temperatures are shown in Figure 6. It can be seen that the grains in the cold-rolling sheet are mainly blue and red, which indicates that it is mainly composed of deformed grains with {111}<uvw> and {001}<uvw> orientations (Figure 6a,e). However, there are more deformed grains with {111}<uvw> orientation in the 90% deformed cold-rolling sheet, and there are some deformed grains with {112}<uvw> orientation. This is because uneven deformation gives the grains different degrees of fragmentation during the cold-rolling process, which produced a lot of orientation gradient area. In addition, shear bands and broken grains preferentially appear in the {111}<uvw> orientation [20], and the addition of nucleation sites is beneficial for grain refinement. Figure 6b–d show that ferrite grains recrystallized after heat treatment. In the 50% reduction cold-rolled annealing plate, grains are mainly randomly distributed, and the number of recrystallized grains in the {111}<uvw> orientation is relatively small. As shown in Figure 6f–h, the crystal orientation in the cold-rolled annealed plate with 90% reduction is mainly composed of {111}<uvw> and partial {112}<uvw> components, and the grain size is small. Ray et al. [21] illustrated that the stored energy (E) in a cold-rolling sheet could be arranged in the order: E_{111}<uvw>_ > E_{112}<uvw>_ > E_{110}<110>_ > E_{110}<001>_. The large cold-rolling reduction can promote the deformed orientation of {111}<uvw>, which provides greater storage energy and driving force for recrystallization. It shows that the orientation of the sheets could be inherited by the cold-rolled and annealed sheets in some extent, and it has a positive influence on the recrystallized grain orientation.

**Figure 7:**
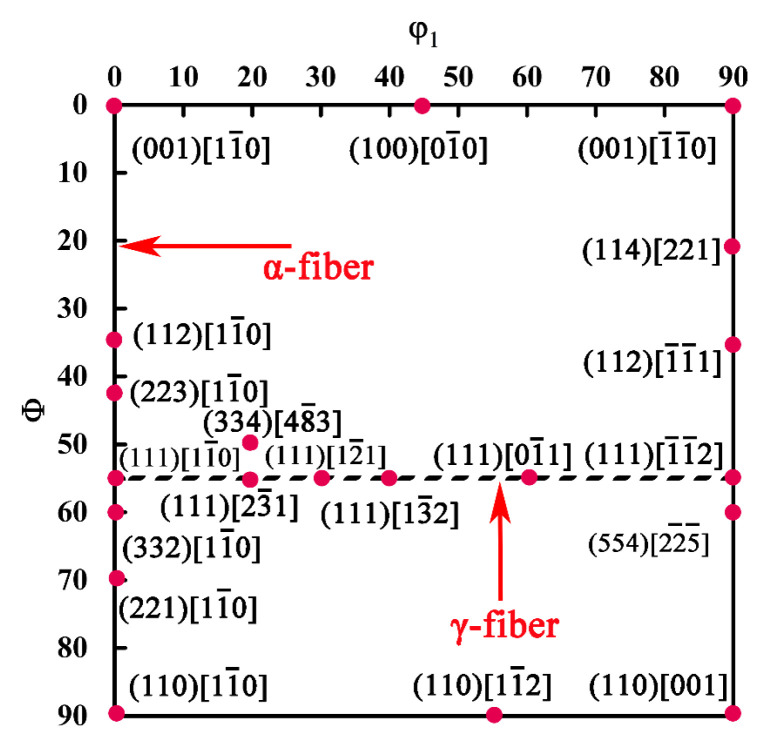
φ2 = 45° ODF sections of the ideal body-centered cubic rolling and recrystallization fibers.

Figure 7 is a schematic of the φ2 = 45° orientation distribution function (ODF) section that contains the texture components discussed in this work. The main texture components in the rolled and annealed ferritic stainless steel sheets are distributed on the α and γ orientation lines. All the orientations that belong to the α-fiber have their <110> axes parallel to the RD, where the main texture components on the α orientation line (φ1 = 0°, Φ = 0~90°, φ2 = 45°) are (001)[1$\bar{1}$0], (112)[1$\bar{1}$0], (223)[1$\bar{1}$0], (332)[1$\bar{1}$0] and (111)[1$\bar{1}$0], etc. In addition, the texture components related to deep drawing properties, e.g., (334)[4$\bar{8}$3] and (554)[2$\bar{2}\bar{5}$], are also easily formed during the annealing process [10].

**Figure 8:**
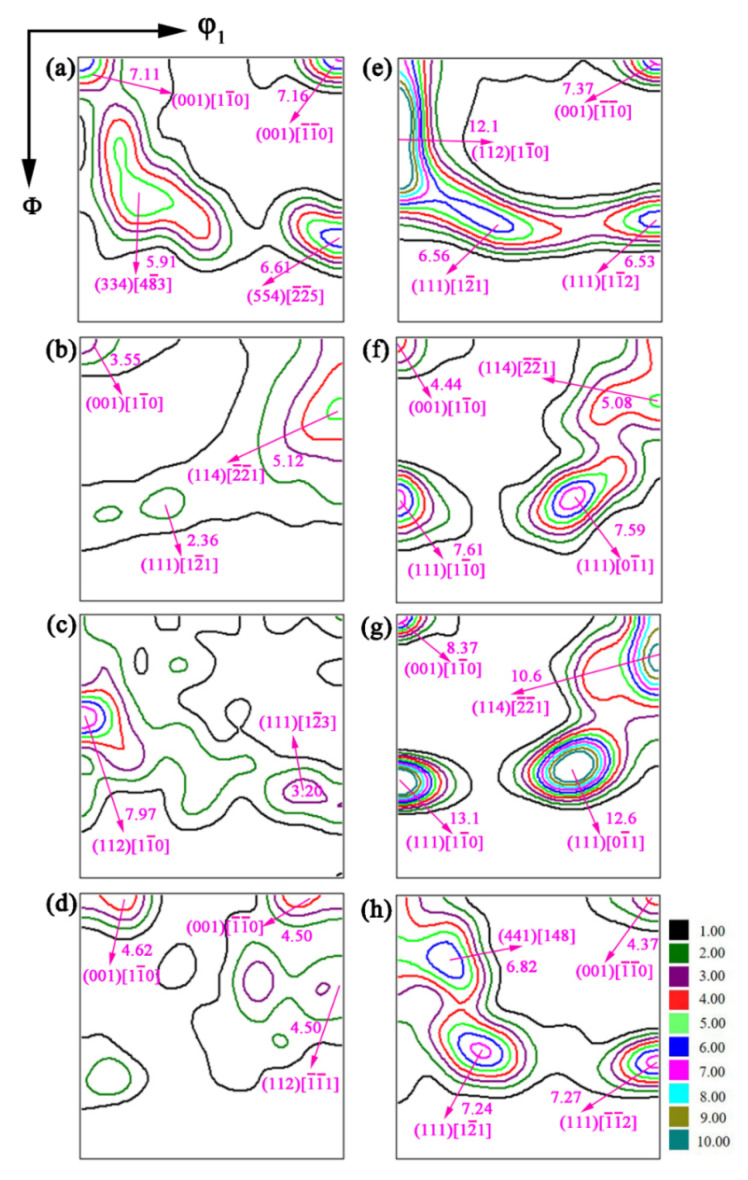
ODFs (φ2 = 45° sections) of X2CrNi12 steel at 50% deformation (**a**–**d**) and 90% deformation (**e**–**h**) with different annealing temperatures, (**a**,**e**) deformed; (**b**,**f**) 720 °C; (**c**,**g**) 740 °C; (**d**,**h**) 770 °C.

The properties of ferritic stainless steel are closely related to the recrystallization texture, and the cold-rolling textures have a great influence on the recrystallization texture [22]. The aggregation state of the grain orientation can be seen from the ODF. The ODFs (φ2 = 45° sections) of specimens at 50% and 90% deformation with different annealing temperatures are reproduced in Figure 8a–h, respectively. The cold-rolling texture of ferritic stainless steels is mainly composed of the weak γ-fiber and strong α-fiber components. Evidently, the specimen of the 50%-deformed mainly contains (001)[1$\bar{1}$0], (001)[$\bar{1}\bar{1}$0], (334)[48$\bar{3}$] and (554)[2$\bar{2}\bar{5}$] components, and the maximum intensity f(g)max = 7.16 (Figure 8a). The specimen of the 50%-720 °C mainly contains the weak α-fiber at (001)[1$\bar{1}$0] orientation and the weak γ-fiber at (111)[12$\bar{1}$] (Figure 8b) orientation. The specimen of the 50%-740 °C mainly contains the strong α-fiber at (112)[1$\bar{1}$0] and the weak γ-fiber at (111)[12$\bar{3}$] (Figure 8c). According to the research results of Raabe et al. [23], {111}<112> and {334}<48$\bar{3}$> have an orientation relationship of 26° <110>, which is close to the orientation relation of 27° <110> of the Σ19a coincidence site lattice (CSL) boundary, and the Ʃ19a grain boundary has a high moving rate. Owing to the high mobility of the Ʃ19a boundary, the {111}<112> nuclei can selectively grow into the {111}<112> texture. The specimen of the 50%-770 °C is mainly composed of weak textures at (001)[1$\bar{1}$0], (001)[$\bar{1}\bar{1}$0] and (112)[1$\bar{1}\bar{1}$] orientations, and the maximum intensity f(g)max = 4.62 (Figure 8d).

When the cold-rolling reduction is 90%, the specimen of the 90%-deformed steel is mainly composed of the strong α-fiber at (112)[1$\bar{1}$0] with f(g)max = 12.1 and the weak γ-fiber at (111)[1$\bar{2}$1] and (111)[1$\bar{1}$2], and the maximum intensity f(g)max = 6.56 (Figure 8e). When the annealing temperature is 720 °C, the textures of (112)[1$\bar{1}$0] and (001)[$\bar{1}\bar{1}$0] disappear, forming the (001)[1$\bar{1}$0] and (114)[$\bar{2}\bar{2}$1] textures (Figure 8f). The α-fiber texture is weakened and the γ-fiber texture is enhanced. The highest intensity among the γ-fiber texture is at {111}〈110〉. As shown in Figure 8g, the texture of the 90%-740 °C steel is consistent with the specimen at 90%-720 °C, but the maximum intensity of the α-fiber texture at (001)[1$\bar{1}$0] increases to 8.37, and the intensity of the γ-fiber textures increase to 13.1 for (111)[1$\bar{1}$0] and 12.6 for (111)[0$\bar{1}$1], respectively. According to the literature, the formation of high-density γ-fiber texture in a stainless steel plate is beneficial to improve the formability of the material [24,25]. As annealing temperature increased to 770 °C, the α-fiber texture disappeared and the strength of the γ-fiber texture decreased gradually. The specimen of the 90%-770 °C is mainly composed of the strong γ-fiber at (111)[1$\bar{2}$1] and (111)[$\bar{1}\bar{1}$2].

**Figure 9:**
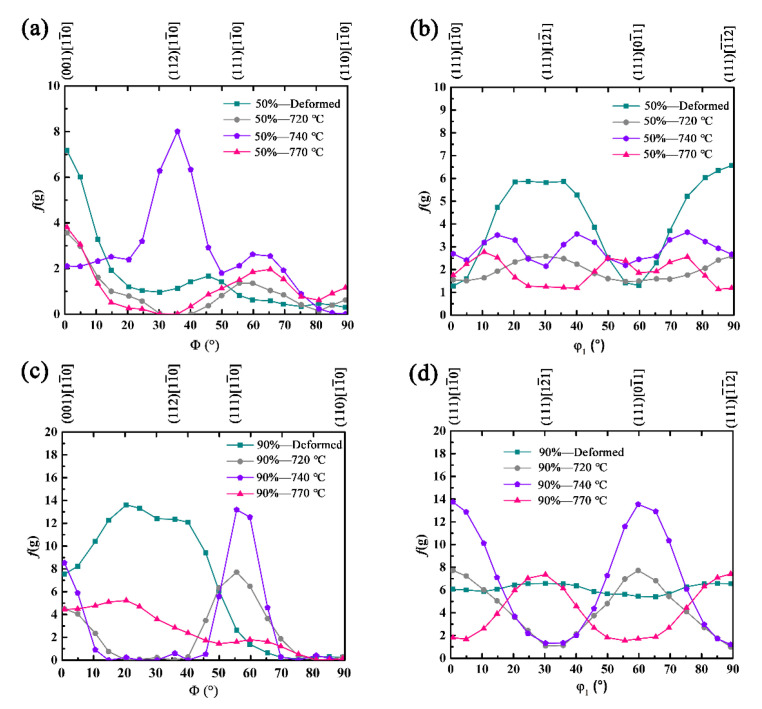
Orientation densities along (**a**,**c**) α-fiber and (**b**,**d**) γ-fiber in the cold-rolled sheets after annealing at different temperatures with (**a**,**b**) 50% reduction and (**c**,**d**) 90% reduction, respectively.

Figure 9 presents the orientation densities of the texture α and γ orientation lines at 50% and 90% cold-rolling reduction with different annealing temperatures. On the whole, the orientation density of the α-fiber and γ-fiber textures is smaller under 50% cold-rolling reduction, a great deal of randomly oriented grains will be produced after annealing, and the overall strength of the texture is low. The α-fiber and γ-fiber textures of cold-rolling sheet at 90% reduction are larger. After the recrystallization annealing, the texture strength increased, and the orientation density of α-fiber and γ-fiber recrystallization texture also improved. When heavily cold-rolling steels is recrystallisation-annealed, the γ-fiber texture is strengthened, while the α-fiber (particularly the {112}<110> component) is decreased. According to the literature [26,27], the density of the α-fiber texture after the recrystallization annealing is weakened at {001}<110> and {112}<110>, γ-fiber texture strength increases, and the maximum orientation density values appear around {111}<112> and {111}<110>. This is basically consistent with the findings of our work.

### 3.3. Grain Boundary Characteristic Distribution

**Figure 10:**
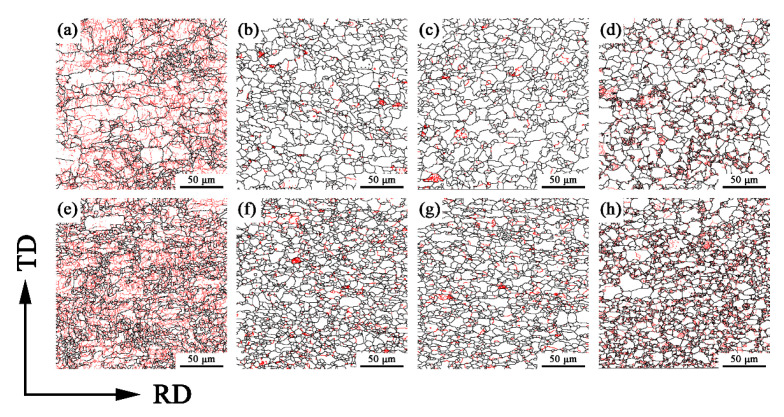
Grain boundary characteristic distribution of X2CrNi12 steel at 50% deformation (**a**–**d**) and 90% deformation (**e**–**h**) with different annealing temperatures, (**a**,**e**) deformed; (**b**,**f**) 720 °C; (**c**,**g**) 740 °C; (**d**,**h**) 770 °C. (The red and black lines represent LAGBs and HAGBs, respectively).

Figure 10 shows the grain boundary characteristic distribution (GBCD) of X2CrNi12 steel obtained with EBSD at 50% and 90% cold-rolling reduction with different annealing temperatures. Geometrically, grain boundaries can be divided into LAGBs, low-Σ CSL boundaries (CSLBs, Σ values are defined as the reciprocal of the ratio of the relocations of two adjacent crystal lattice arrays) and general HAGBs (or high-Σ CSL grain boundaries) according to the crystallographic orientation relationship between adjacent grains [28]. After cold-rolling deformation, the grains are broken and a large number of subgrains will be generated, resulting in small orientation difference between two adjacent grains, and more LAGBs are displayed when calibrated by EBSD data processing software (Figure 10a,e). Compared with the cold-rolling sheet, the amount of LAGBs in the annealed sheet decreases due to the increase of grain size of ferrite and the decrease of grain boundaries, and the dominant grain boundary in polycrystalline sheet is HAGBs (Figure 10b–d,f–h). It is worth noting that when the annealing temperature is 770 °C, a large number of low-angle grain boundaries are distributed on martensite, indicating that the grain boundaries in martensite are mainly low-angle grain boundaries. According to the research results presented by Lu et al. [29], Σ3 and Σ7 grain boundaries are dominant in martensitic structure and the interface energy is low.

**Figure 11:**
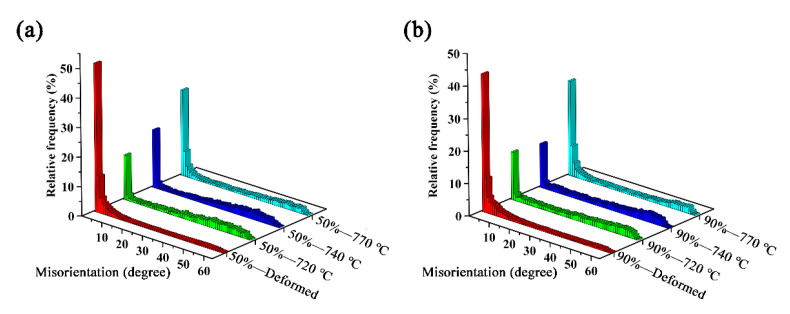
Misorientation distributions of X2CrNi12 steel at 50% deformation (**a**) and 90% deformation (**b**) with different annealing temperatures.

**Figure 12:**
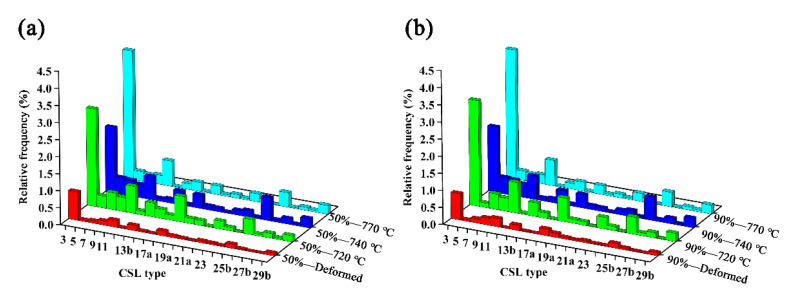
Low-Σ CSL boundary distributions of X2CrNi12 steel at 50% deformation (**a**) and 90% deformation (**b**) with different annealing temperatures.

In order to display the frequency distribution of LAGBs and HAGBs of different specimens more intuitively, the frequency distribution of misorientation distributions of X2CrNi12 steels are shown in Figure 11, and Table 2 lists their corresponding values. It is obvious that the frequency of LAGBs in specimens of 50%-770 °C and 90%-770 °C are 51% and 53%, respectively, which are the largest among the annealed specimens. In addition, it can be seen that more LAGBs can be obtained in the recrystallized specimens under 90% cold-rolling reduction. Additionally, the low-Σ CSL boundaries distributions of specimens at 50% deformation and 90% deformation with different annealing temperatures are shown in Figure 12, and Table 2 lists their corresponding values. On the whole, the frequency of low-Σ CSLs increases greatly after annealing treatment. In the 50% cold-rolled and annealed steel plate, the maximum frequency of low-Σ CSLs is 8.15% in the grain boundary of 50%-720 °C. The maximum frequency of low-Σ CSLs in the grain boundary of 90%-740 °C is 9.09%.

### 3.4. Electrochemical Test

**Figure 13:**
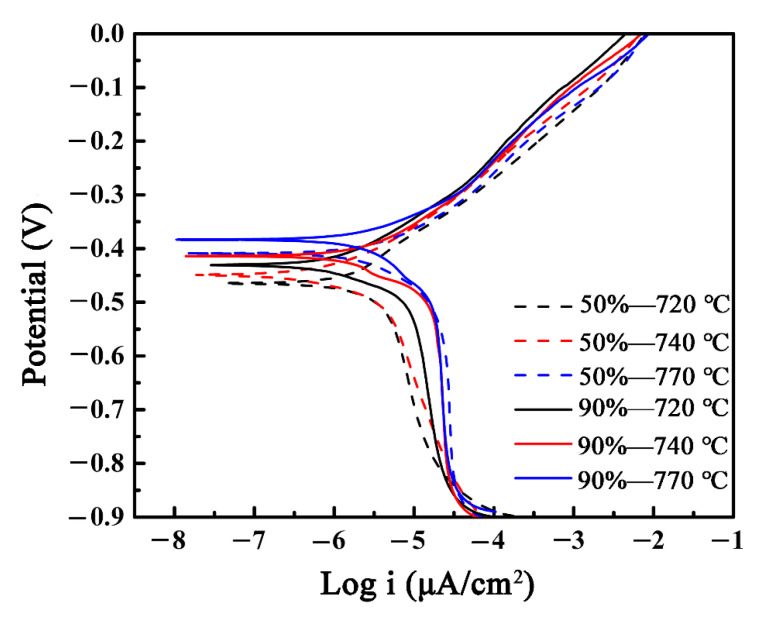
Potentiodynamic polarization curves of the experimental steels in 3.5 wt.% NaCl solution.

To analyze the effect of cold-rolling reduction on the corrosion resistance properties of recrystallized X2CrNi12 ferritic stainless steel, an electrochemical analysis was carried out. Figure 13 shows the potentiodynamic polarization curves of specimens at 50% and 90% deformation reduction with different annealing temperatures in the 3.5 wt.% NaCl solution. According to the polarization curve, the corrosion current density (i_corr_) and corrosion potential (E_corr_) can be calculated (Table 3 and Table 4). The corrosion current densities of the 50%-720 °C, 50%-740 °C and 50%-770 °C are 5.613, 3.269 and 2.433 μA/cm^2^, respectively. The values can be used to assess the active dissolution ability of the specimens [30,31].

Therefore, in the annealed steels sheet with a cold-rolling reduction of 50%, the specimen of 50%-770 °C exhibits the best corrosion resistance. Additionally, the corrosion current densities of the 90%-720 °C, 90%-740 °C and 90%-770 °C are 3.71, 2.81 and 2.01 μA/cm^2^, respectively. Meanwhile the specimen of 90%-770 °C exhibits the best corrosion resistance. Additionally, the corrosion potentials of the specimens at 50%-770 °C and 90%-770 °C are −0.408 V and −0.381 V, respectively. The recrystallization specimens of X2CrNi12 stainless steel obtained better corrosion resistance when the cold-rolling reduction was 90%, and the corrosion properties of cold-rolling steel plates with different reductions are the best when annealed at 770 °C. In addition, no passivation zones were found in the polarization curves of the specimens. This is because the Cr content in X2CrNi12 stainless steel is relatively low; the presence of Cl^−^ makes it difficult to passivate the ferritic stainless steel with weak passivation ability, and directly enters the stage of high corrosion current at a higher potential.

**Figure 14:**
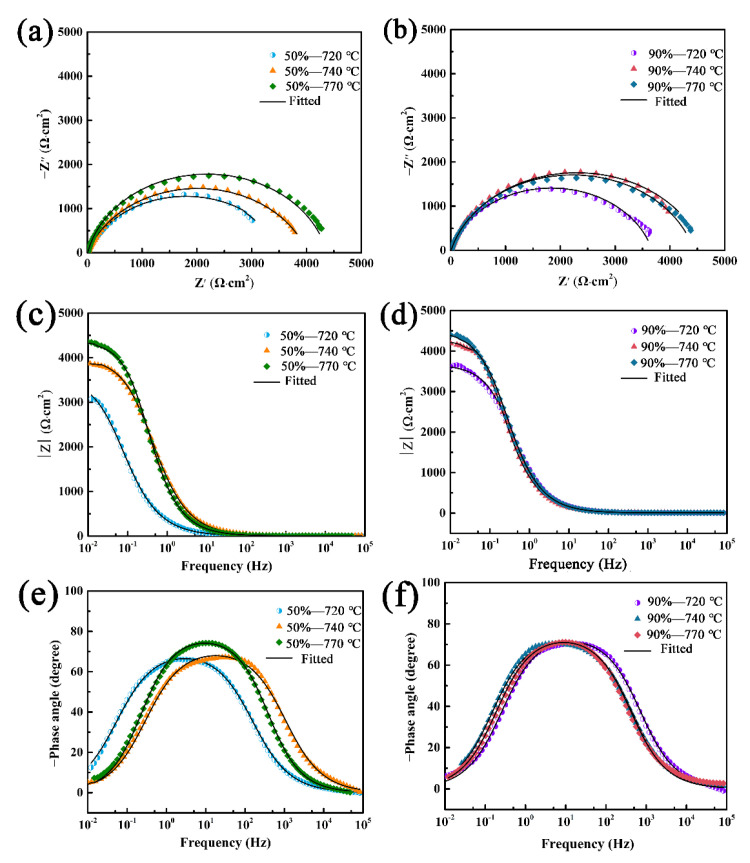
EIS plots of X2CrNi12 specimens with 50% and 90% cold-rolling reduction at different annealing temperatures: (**a**,**b**) Nyquist plots, (**c**,**d**) Bode impendence plots, (**e**,**f**) Bode phase angle plots.

Figure 14 shows the EIS curves of X2CrNi12 stainless steel specimens at 50% and 90% cold-rolling reduction with different annealing temperatures. According to the Nyquist plots (Figure 14a,b), it can be seen that all the specimens are composed of a high-frequency capacitor circuit. The larger the radius of the capacitor ring, the greater the transfer resistance and the better the corrosion resistance [6]. The specimen has the greater transfer resistance and better corrosion resistance when annealed at 90% cold-rolling reduction compared with 50% cold-rolling reduction. Figure 14c,d are the Bode impendence plots, and Figure 14e,f are the Bode phase angle plots. In the Bode impedance plots, the greater the impedance modulus, the greater the polarization resistance and the better the corrosion resistance [6]. In the Bode phase angle plots, the larger the peak and peak width of the phase angle, the greater the resistance and the better the corrosion performance [6]. The results show that the specimens have larger phase angle peaks, peak width and maximum impedance modulus when annealed at 90% cold-rolling reduction, which indicates that the specimens have better corrosion resistance.

**Figure 15:**
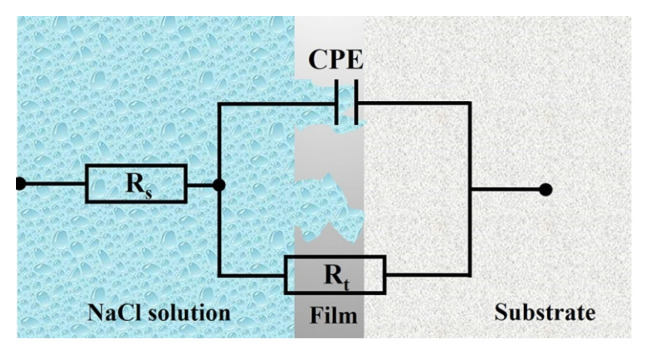
Equivalent circuits used for fitting the EIS.

The equivalent circuit diagram of the X2CrNi12 stainless steel with 50% and 90% cold-rolling reduction at different annealing temperatures is shown in Figure 15. In the equivalent circuit diagram, R_s_ is the solution resistance between the working electrode and the reference electrode, and its value only depends on the conductivity of the test medium; R_t_ is the polarization resistance, and the larger the R_t_ value, the better the corrosion resistance; CPE is the constant phase element, and it is mainly a double-layer capacitance on the surface of the electrolyte/substrate [32]. The CPE consists of two parameters, Y_0_ and n. Y_0_ represents the non-ideal capacitance, which is a dispersion effect caused by cracks, surface oxide films, impurities and secondary phases [33]; n (0 < n < 1) is a dispersion degree index. When n is equal to 0, CPE is regarded as a pure resistance, and when n is equal to 1, CPE is regarded as a pure capacitance [34]. The corresponding electrochemical parameters of the equivalent circuit diagram are shown in Table 5. It can be seen that the specimen has a larger R_t_ value when annealed at 90% cold-rolling reduction, indicating that its corrosion rate is lower and its corrosion resistance is better. In addition, under the same cold-rolling reduction, the specimen has the largest R_t_ when annealed at 770 °C, indicating that its corrosion rate is the lowest and the corrosion resistance is the best. Therefore, the specimen of 90%-770 °C exhibits the best corrosion resistance.

According to the above analysis, when the cold-rolling reduction of X2CrNi12 stainless steel is 90%, the recrystallized specimen has a smaller grain size, and grain refinement provides better conditions for the formation of passivation film on the stainless steel surface, which is conducive to the growth of thicker passivation film with fewer defects, improving the corrosion resistance of the matrix [35]. On the other hand, increasing the proportion of special grain boundaries (LAGBs and low-Σ CSL boundaries) also improved the corrosion resistance of the material [36]. LAGBs and low-Σ CSL boundaries have higher structural order, smaller free volume, lower interface energy, and stronger grain boundary corrosion resistance than HAGBs and high-Σ CSL boundaries [37]. It can be seen from Table 2 that the proportion of special boundaries in the grain boundary distribution of recrystallized X2CrNi12 stainless steel specimen is higher when the cold-rolling reduction is 90%. The corrosion properties of cold-rolling steel sheets are the best when annealed at 770 °C with different reductions. With the increase of annealing temperature, the precipitated (Fe, Cr)_23_C_6_ content decreases. The microstructures of the 50%-770 °C and 90%-770 °C samples are composed of ferrite, martensite and a minimal amount of (Fe, Cr)_23_C_6_. The precipitation of (Fe, Cr)_23_C_6_ consumes the Cr in the matrix. This could reduce the corrosiveness of the steel in Cl^−^ environment, is prone to pitting corrosion and reduces the formation of Cr-rich passivation film on the surface [6]. In addition, the proportion of LAGBs in martensite is greater, and the formation of the appropriate amount of martensite can greatly increase the LAGBs in the matrix, thus improving the corrosion resistance of X2CrNi12 stainless steel.

## 4. Conclusions

(1) The crystal orientation characteristics of the cold-rolled sheet can be inherited by the cold-rolled and annealed sheet, and a large cold-rolling reduction can increase the {111}<uvw> texture in the cold-rolling sheet, which can reduce the recrystallized grain size.
(2) The orientation density of α and γ fibers is small at 50% cold-rolling reduction, and a large number of randomly oriented grains will be generated after recrystallization annealing, and the overall texture strength is low. When 90% cold-rolling steel is recrystallisation annealed, the γ-fiber texture at {111}<110> is strengthened and the α-fiber, particularly the {112}<110> component is decreased, which is beneficial to improve the formability of the steels.
(3) The proportions of the special boundaries (LAGBs and Low-Σ CSL boundaries) are higher when the cold-rolling reduction is 90%, especially when the annealing temperature is 770 °C. Additionally, the LAGBs and low-Σ CSLs are 53% and 7.43%, respectively, showing the best corrosion resistance.

## Figures and Tables

**Table 1:** Composition of experimental steel (wt.%).

C	Mn	Si	Cr	P	S	Ni	N	Fe
0.02	1.2	0.28	11.7	0.02	0.01	0.68	0.013	Bal.

**Table 2:** Grain boundary distribution of X2CrNi12 steel specimens.

Specimens	HAGBs	LAGBs	Low-Σ CSLs
50%-deformed	15%	85%	2.05%
50%-720 °C	75%	25%	8.15%
50%-740 °C	72%	28%	6.86%
50%-770 °C	49%	51%	7.50%
90%-deformed	20%	80%	2.32%
90%-720 °C	69%	31%	7.98%
90%-740 °C	71%	29%	9.09%
90%-770 °C	47%	53%	7.43%

**Table 3:** i_corr_, E_corr_ values of specimens at 50% deformation with different annealing temperatures derived from the potentiodynamic curves.

Specimens	50%-720 °C	50%-740 °C	50%-770 °C
i_corr_ (μA/cm^2^)	5.613 ± 0.432	3.269 ± 0.485	2.433 ± 0.211
E_corr_ (V_SCE_)	−0.463 ± 0.013	−0.449 ± 0.015	−0.408 ± 0.092

**Table 4:** i_corr_, E_corr_ values of specimens at 90% deformation with different annealing temperatures derived from the potentiodynamic curves.

Specimens	90%-720 °C	90%-740 °C	90%-770 °C
i_corr_ (μA/cm^2^)	3.71 ± 0.672	2.81 ± 0.563	2.01 ± 0.232
E_corr_ (V_SCE_)	−0.430 ± 0.015	−0.413 ± 0.012	−0.381 ± 0.027

**Table 5:** Electrochemical parameters of the fitting equivalent circuits.

Specimens	R_s_	R_t_	CPE	
	Ω·cm^2^	Ω·cm^2^	Y_0_ (μF/cm^2^)	n
50%-720 °C	7.152	4985	330.5	0.8631
50%-740 °C	7.289	5026	285.6	0.8725
50%-770 °C	7.891	6446	112.8	0.8847
90%-720 °C	7.289	5103	300.1	0.8701
90%-740 °C	7.446	5687	245.7	0.8795
90%-770 °C	7.986	6852	103.5	0.8912

## Data Availability

The data presented in this study are available on request from the corresponding author.

## References

[1] Topic M., Allen C., Tait R. (2007). The effect of cold work and heat treatment on the fatigue behaviour of 3CR12 corrosion resistant steel wire. Int. J. Fatigue.

[2] Zheng H., Ye X., Jiang L., Wang B., Liu Z., Wang G. (2010). Study on microstructure of low carbon 12% chromium stainless steel in high temperature heat-affected zone. Mater. Des..

[3] Zheng H., Ye X., Li J., Jiang L., Liu Z., Wang G., Wang B. (2010). Effect of carbon content on microstructure and mechanical properties of hot-rolled low carbon 12Cr–Ni stainless steel. Mater. Sci. Eng. A.

[4] Wang L.-X., Song C.-J., Sun F.-M., Li L.-J., Zhai Q.-J. (2009). Microstructure and mechanical properties of 12 wt.% Cr ferritic stainless steel with Ti and Nb dual stabilization. Mater. Des..

[5] Zhang Z., Wang Z., Wang W., Yan Z., Dong P., Du H., Ding M. (2015). Microstructure evolution in heat affected zone of T4003 ferritic stainless steel. Mater. Des..

[6] Li R., Fu B.-G., Dong T.-S., Li G.-L., Li J.-K., Zhao X.-B., Liu J.-H. (2022). Effect of annealing treatment on microstructure, mechanical property and anti-corrosion behavior of X2CrNi12 ferritic stainless steel. J. Mater. Res. Technol..

[7] Park Y., Lee D., Gottstein G. (1996). Development of texture inhomogeneity during hot rolling in interstitial free steel. Acta Mater..

[8] Cai G., Li C., Wang D., Zhou Y. (2018). Investigation of annealing temperature on microstructure and texture of Fe-19Cr-2Mo-Nb-Ti ferritic stainless steel. Mater. Charact..

[9] Fu J., Li F., Sun J., Wu Y. (2018). Texture, orientation, and mechanical properties of Ti-stabilized Fe-17Cr ferritic stainless steel. Mater. Sci. Eng. A.

[10] de Abreu H., Bruno A., Tavares S., Santos R., Carvalho S. (2006). Effect of high temperature annealing on texture and microstructure on an AISI-444 ferritic stainless steel. Mater. Charact..

[11] Huh M.-Y., Engler O. (2001). Effect of intermediate annealing on texture, formability and ridging of 17% Cr ferritic stainless steel sheet. Mater. Sci. Eng. A.

[12] Zhang C., Liu Z., Wang G. (2011). Effects of hot rolled shear bands on formability and surface ridging of an ultra purified 21% Cr ferritic stainless steel. J. Mater. Process. Technol..

[13] Rodrigues D.G., Alcântara C.M., Oliveira T.R., Gonzalez B.M. (2019). The effect of grain size and initial texture on microstructure, texture, and formability of Nb stabilized ferritic stainless steel manufactured by two-step cold rolling. J. Mater. Res. Technol..

[14] Yan H.T., Bi H.Y., Li X., Xu Z. (2009). Effect of two-step cold rolling and annealing on texture, grain boundary character distribution and r-value of Nb + Ti stabilized ferritic stainless steel. Mater. Charact..

[15] Han J., Li H.J., Zhu Z.X., Jiang L.Z., Xu H.G., Ma L. (2014). Effects of processing optimisation on microstructure, texture, grain boundary and mechanical properties of Fe-17Cr ferritic stainless steel thick plates. Mater. Sci. Eng. A.

[16] Shimada M., Kokawa H., Wang Z., Sato Y., Karibe I. (2002). Optimization of grain boundary character distribution for intergranular corrosion resistant 304 stainless steel by twin-induced grain boundary engineering. Acta Mater..

[17] Li J., Ren X., Gao X. (2020). Effect of superplastic deformation on microstructure evolution of 3207 duplex stainless steel. Mater. Charact..

[18] Mola J., Jung I., Park J., Chae D., De Cooman B.C. (2012). Ridging Control in Transformable Ferritic Stainless Steels. Met. Mater. Trans. A.

[19] Meng L., Lu H., Li W., Guo H., Tian J., Liang W. (2021). High strength and plasticity of AISI 430 ferritic stainless steel achieved by a recrystallization annealing before quenching and partitioning process. Mater. Sci. Eng. A.

[20] Barnett M.R. (1998). Role of in-grain shear bands in the nucleation of <111>//ND recrystallization textures in warm rolled steel. ISIJ Int..

[21] Ray R.K., Jonas J.J., Hook R.E. (1994). Cold rolling and annealing textures in low carbon and extra low carbon steels. Int. Mater. Rev..

[22] Raabe D. (1996). On the influence of the chromium content on the evolution of rolling textures in ferritic stainless steels. J. Mater. Sci..

[23] Raabe D., Lüucke K. (1993). Textures of Ferritic Stainless Steels. Mater. Sci. Technol..

[24] Miyamoto H., Xiao T., Uenoya T., Hatano M. (2010). Effect of Simple Shear Deformation Prior to Cold Rolling on Texture and Ridging of 16% Cr Ferritic Stainless Steel Sheets. ISIJ Int..

[25] Ma X., Zhao J., Du W., Zhang X., Jiang L., Jiang Z. (2017). An analysis of ridging of ferritic stainless steel 430. Mater. Sci. Eng. A.

[26] Hamada J.-I., Ono N., Inoue H. (2011). Effect of Texture on *r*-value of Ferritic Stainless Steel Sheets. ISIJ Int..

[27] Bai Y., He T., Liu Y. (2018). Effects of Sn microalloying on cold rolling and recrystallization textures and microstructure of a ferritic stainless steel. Mater. Charact..

[28] Liu M., Gong W., Zheng R., Li J., Zhang Z., Gao S., Ma C., Tsuji N. (2022). Achieving excellent mechanical properties in type 316 stainless steel by tailoring grain size in homogeneously recovered or recrystallized nanostructures. Acta Mater..

[29] Lu H.-H., Li W.-Q., Du L.-Y., Guo H.-K., Liang W., Zhang W.-G., Liu Z.-G. (2019). The effects of martensitic transformation and (Fe, Cr)23C6 precipitation on the properties of transformable ferritic stainless steel. Mater. Sci. Eng. A.

[30] Meng G., Li Y., Shao Y., Zhang T., Wang Y., Wang F., Cheng X., Dong C., Li X. (2015). Effect of Microstructures on Corrosion Behavior of Nickel Coatings: (II) Competitive Effect of Grain Size and Twins Density on Corrosion Behavior. J. Mater. Sci. Technol..

[31] Fu Y., Liu C., Hao H., Xu Y.-D., Zhu X.-R. (2021). Effect of ageing treatment on microstructures, mechanical properties and corrosion behavior of Mg-Zn-RE-Zr alloy micro-alloyed with Ca and Sr. China Foundry.

[32] Hu Z., Yin Z., Yin Z., Wang K., Liu Q., Sun P., Yan H., Song H., Luo C., Guan H. (2020). Corrosion behavior characterization of as extruded Mg-8Li-3Al alloy with minor alloying elements (Gd, Sn and Cu) by scanning Kelvin probe force microscopy. Corros. Sci..

[33] Liu X., Xue J., Liu S. (2018). Discharge and corrosion behaviors of the α-Mg and β-Li based Mg alloys for Mg-air batteries at different current densities. Mater. Des..

[34] Mahdavian M., Attar M. (2006). Another approach in analysis of paint coatings with EIS measurement: Phase angle at high frequencies. Corros. Sci..

[35] Fattah-Alhosseini A., Vafaeian S. (2016). Influence of grain refinement on the electrochemical behavior of AISI 430 ferritic stainless steel in an alkaline solution. Appl. Surf. Sci..

[36] Ishibashi R., Horiuchi T., Kuniya J., Yamamoto M., Tsurekawa S., Kokawa H., Watanabe T., Shoji T. (2005). Effect of Grain Boundary Character Distribution on Stress Corrosion Cracking Behavior in Austenitic Stainless Steels. Mater. Sci. Forum.

[37] Pan Y., Adams B., Olson T., Panayotou N. (1996). Grain-boundary structure effects on intergranular stress corrosion cracking of alloy X-750. Acta Mater..

